# Ultrasound-Guided Hydrodissection of the Thoracodorsal Nerve and Axillary Nerve in a Gymnast With Shoulder Pain Associated With Superior Labral Anterior-Posterior Lesions: A Case Report

**DOI:** 10.7759/cureus.60157

**Published:** 2024-05-12

**Authors:** Toru Omodani

**Affiliations:** 1 Orthopaedics, Tokyo Advanced Orthopaedics, Tokyo, JPN

**Keywords:** superior labral anterior-posterior lesions, gymnast, axillary nerve, thoracodorsal nerve, hydrodissection, ultrasound

## Abstract

A 20-year-old male national-level gymnast presented with left shoulder pain attributed to a superior labral anterior-posterior (SLAP) lesion. Physical examination revealed pain in the anterosuperior area at maximum shoulder elevation, with a positive combined abduction test and horizontal flexion test indicating a restriction in glenohumeral joint motion. Rather than directly addressing the SLAP lesion, ultrasound-guided hydrodissections of the thoracodorsal and axillary nerves were performed, leading to immediate alleviation of pain and mobility constraints. This innovative approach, emphasizing shoulder function, offers a novel therapeutic strategy for SLAP-associated shoulder pain in athletes.

## Introduction

Superior labral anterior-posterior (SLAP) lesions are one of the overuse injuries that occur in the shoulder joints of athletes [1]. Symptoms of SLAP lesions commonly include pain during shoulder elevation in activities such as gymnastics on the rings and baseball pitching [2,3]. Initial treatments often include rest, medication, injections, and rehabilitation. It has been reported that the completion of rehabilitation programs aimed at improving shoulder joint flexibility and strengthening the rotator cuff and core muscles results in an approximately 80% rate of return to sports [4]. If conservative treatments are not effective, arthroscopic surgery is performed for debridement or suturing of the SLAP lesion [5]. After arthroscopic surgery, the average rate of return to sports is 70%, with the reported average duration until return being nine months [6].

To date, there have been no reports of hydrodissection targeting nerves for pain associated with SLAP lesions. We report a case where hydrodissection of the thoracodorsal nerve and axillary nerve was effective for shoulder pain associated with SLAP lesions.

## Case presentation

A 20-year-old male gymnast at the national competition level began experiencing left shoulder pain during competitions. The main complaint was pain during the giant swing on the rings, which presented as a chronic onset without any clear trauma. After undergoing a plain MRI at a nearby medical facility, no obvious abnormalities were noted. However, as the pain did not improve despite rehabilitation, the patient visited my clinic three months after the onset of symptoms.

**Figure 1:**
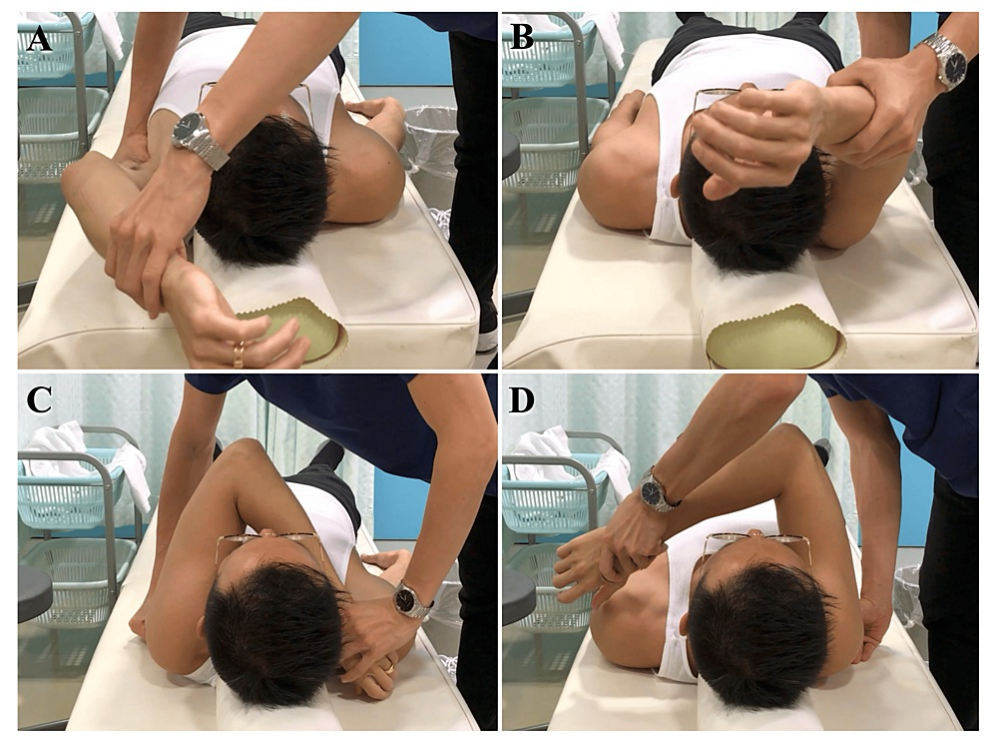
CAT and HFT (A) For the CAT, the scapula is manually fixed, and flexion of the shoulder joint is performed. (B) If the range of motion is reduced compared to the healthy side, it is determined to be positive. (C) For the HFT, the scapula is manually fixed, and horizontal flexion of the shoulder joint is performed. (D) If the range of motion is reduced compared to the healthy side, it is determined to be positive. Note: The images provided are not from this case but are reference images from another patient. CAT, combined abduction test; HFT, horizontal flexion test

**Figure 2:**
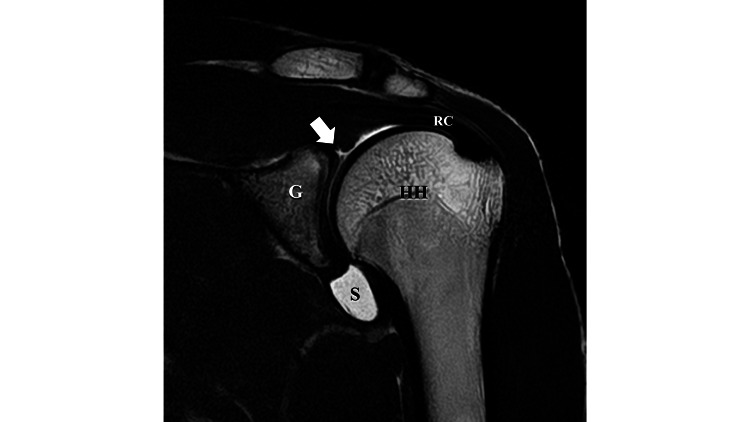
Contrast-enhanced MRI A SLAP lesion (indicated by the arrow) was observed. G, glenoid cavity of the scapula; HH, humeral head; RC, rotator cuff; S, saline injected into the joint for contrast; SLAP, superior labral anterior-posterior

During the physical examination, pain was observed in the anterosuperior area at maximum shoulder elevation. The O'Brien test, which is considered effective for detecting SLAP lesions, was positive [7]. The combined abduction test (CAT) and horizontal flexion test (HFT) were positive, suggesting a restriction in the range of motion of the glenohumeral joint (Figure 1). Contrast-enhanced MRI revealed a SLAP lesion that is believed to be Type 2 (Figure 2). In the CAT test, which involves forward elevation of the shoulder joint, the thoracodorsal nerve located diagonally below the glenohumeral joint was most stretched, while in the HFT test, which involves horizontal adduction of the shoulder joint, the axillary nerve located diagonally behind the glenohumeral joint was most stretched. It is hypothesized that these positions contribute to the tightness of the glenohumeral joint. Considering that the symptoms of the SLAP lesion, accompanied by shoulder joint mobility restriction, were due to the involvement of the thoracodorsal nerve and axillary nerve, we decided to perform hydrodissection on these nerves.

**Figure 3:**
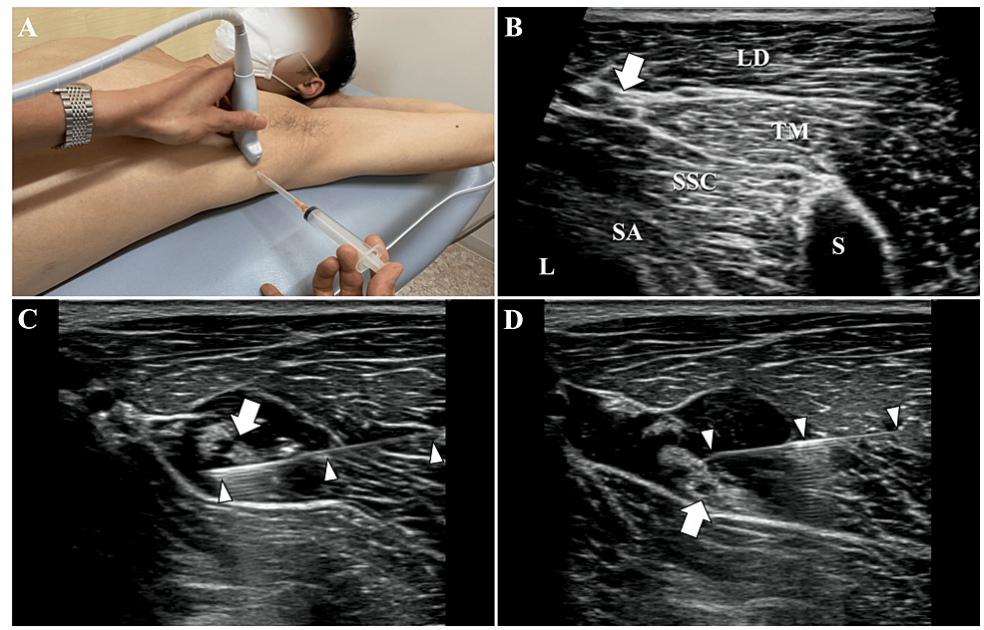
Hydrodissection of the thoracodorsal nerve (A) Positioning of the probe and direction of needle insertion. (B) In the ultrasound image, the neurovascular bundle, which includes the thoracodorsal nerve and thoracodorsal artery, was observed to traverse the space formed by the latissimus dorsi, teres major, and subscapularis muscles. (C, D) The needle was inserted into the deep (C) and superficial (D) layers of the thoracodorsal nerve, and hydrodissection was performed by fluid separation from the surrounding tissues. The arrows indicate the neurovascular bundle containing the thoracodorsal nerve, while the arrowheads indicate the needles. L, lung; LD, latissimus dorsi; S, scapula; SA, serratus anterior; SSC, subscapularis; TM, teres major

The patient was positioned supine on the bed, with the shoulder elevated. The ultrasound probe was placed in the short-axis orientation over the latissimus dorsi muscle (Figure 3A). Using the thoracodorsal artery, which runs between the fascia of the latissimus dorsi, teres major, and subscapularis muscles, as a landmark, hydrodissection of the thoracodorsal nerve was performed (Figure 3B, 3C, 3D). Using 5 ml of 0.09% lidocaine diluted with saline, the thoracodorsal nerve and surrounding tissue were hydrodissected to create a fluid separation. Immediately after the injection, the CAT turned negative.

**Figure 4:**
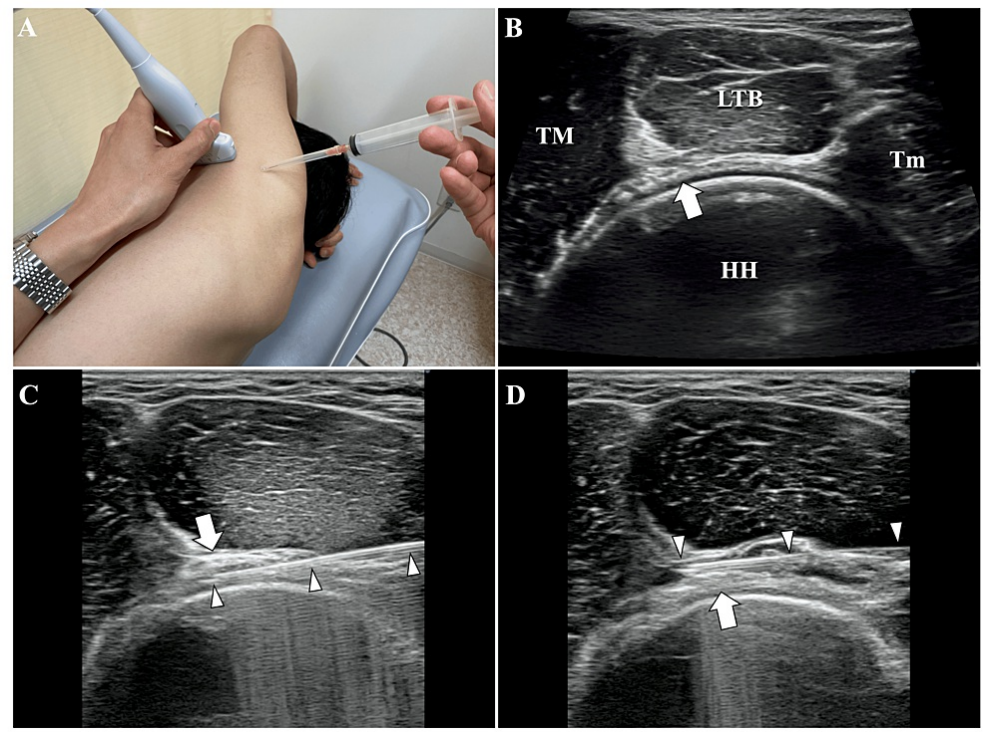
Hydrodissection of the axillary nerve (A) Positioning of the probe and direction of needle insertion. (B) In the ultrasound image, the neurovascular bundle, which includes the axillary nerve and axillary artery, was observed to traverse the space formed by the humeral head, teres major, long head of the triceps brachii, and teres minor muscles. (C, D) The needle was inserted into the deep (C) and superficial (D) layers of the axillary nerve, and hydrodissection was performed by fluid separation from the surrounding tissues. The arrows indicate the neurovascular bundle containing the thoracodorsal nerve, while the arrowheads indicate the needles. HH, humeral head; LTB, long head of triceps brachii; Tm, teres minor; TM, teres major

Next, the patient’s position was changed to lateral decubitus. The probe was placed in the short-axis orientation in the axilla relative to the upper arm (Figure 4A). Using the axillary artery, which runs through the space formed by the humeral head, teres major, long head of the triceps brachii, and teres minor, as a landmark, hydrodissection of the axillary nerve was performed (Figure 4B, 4C, 4D). Using 5 ml of 0.09% lidocaine diluted with saline, the axillary nerve and surrounding tissue were hydrodissected to create a fluid separation. Immediately after the injection, the HFT turned negative.

After these hydrodissections, the pain during maximum shoulder elevation disappeared. There was no pain even when performing the giant swing on the rings, and the athlete’s performance returned to the level before the onset of shoulder pain. In the one-year follow-up post-treatment, the patient experienced no pain during maximum shoulder elevation, and both CAT and HFT were negative. There was no recurrence of shoulder pain during the competition. He was able to perform all six gymnastic events, including the giant swing on the rings, at a national competition level, just as he did before the onset of pain.

## Discussion

In this case, pain relief was achieved for shoulder pain associated with SLAP lesions by performing a hydrodissection of the thoracodorsal nerve and axillary nerve. The novelty and significance of this report lie in the treatment focusing on the thoracodorsal nerve and axillary nerve rather than on the SLAP lesion itself.

While SLAP lesions have a high prevalence among elite gymnasts, many are also believed to be asymptomatic [8]. It is believed that the onset of SLAP lesions is related to shoulder joint dysfunction, and treatments such as rehabilitation aimed at functional improvement are considered important [9]. In this case, both CAT and HFT were positive. CAT and HFT are tests performed while the patient is supine on the bed, with the scapula manually stabilized, and involve flexion or horizontal flexion of the shoulder joint. It is believed that the CAT detects tightness in the inferior aspect of the glenohumeral joint, while the HFT detects tightness in the posteroinferior aspect [10]. When there is tightness in the inferior or posterior aspect of the shoulder joint, it is believed that the centricity of the humeral head relative to the glenoid fossa changes during shoulder elevation [11]. Under such circumstances with obligate translation, the SLAP is more susceptible to strain [12]. In this case, it was speculated that the range of motion limitation of the glenohumeral joint, stemming from the tightness in the posteroinferior aspect of the shoulder joint, increased the strain on the SLAP, leading to its symptomatic manifestation. CAT and HFT are considered tests for detecting tightness in the scapulohumeral joint. However, they are not specialized tests for detecting SLAP lesions, and their specificity and sensitivity have not been verified in the literature.

The hydrodissection of the thoracodorsal nerve and the axillary nerve resulted in the CAT and HFT becoming negative, and the pain during competition disappeared. Hydrodissection refers to a technique of fluidic dissection between tissues using a medicinal solution, and hydrodissection specifically targeting nerves is called nerve hydrodissection [13,14]. Biomechanical studies have shown that performing hydrodissection on nerves improves their gliding resistance [15]. In addition to reducing the gliding resistance of nerves, hydrodissection is believed to be effective in alleviating pain originating from nerves by improving local circulation around the nerve [16]. The positive CAT and HFT in this case might have reflected the entrapment syndrome of the thoracodorsal nerve and axillary nerve, respectively. The fact that CAT and HFT turned negative immediately after hydrodissection might suggest that one of the mechanisms was the resolution of the nerve entrapment syndrome. It is thought that the pain disappeared as a result of the reduced strain on the SLAP lesions, due to the resolution of the obligate translation after the improvement in the range of motion of the shoulder joint. Of course, in this case, the SLAP lesion itself was not repaired. However, it is believed that many high-level gymnasts carry SLAP lesions asymptomatically. Therefore, the treatment was conducted not so much to repair the structural failure but rather to improve the function of the glenohumeral joint to prevent symptoms. However, it is possible that the SLAP lesion could worsen in the future, reaching a point where conservative treatment may no longer be sufficient.

## Conclusions

This case report demonstrated that pain associated with SLAP lesions in a gymnast was alleviated by performing hydrodissection of the thoracodorsal nerve and axillary nerve. The novelty of this report lies in the fact that instead of directly addressing the SLAP lesion itself, we focused on the function of the shoulder joint and performed injections targeting the nerves under ultrasound guidance. The ultrasound-guided hydrodissection of the thoracodorsal nerve and axillary nerve has been suggested as a potentially effective treatment option for shoulder pain associated with SLAP lesions.
